# Neutralizing Antibodies to Severe Fever with Thrombocytopenia Syndrome Virus 4 Years after Hospitalization, China

**DOI:** 10.3201/eid2211.160414

**Published:** 2016-11

**Authors:** Yu-ting Huang, Li Zhao, Hong-ling Wen, Yi Yang, Hao Yu, Xue-jie Yu

**Affiliations:** Shandong University, Jinan, China (Y.-T. Huang, L. Zhao, H.-L. Wen, Y. Yang, X.-J. Yu);; Fudan University, Shanghai, China (H. Yu);; University of Texas Medical Branch, Galveston, Texas, USA (X.-J. Yu)

**Keywords:** severe fever with thrombocytopenia syndrome, SFTS, severe fever with thrombocytopenia syndrome virus, SFTSV, viruses, bunyavirus, hemorrhagic fever, neutralizing antibodies, plaque assay, China

## Abstract

Severe fever with thrombocytopenia syndrome is an emerging hemorrhagic fever disease in eastern Asia, caused by a tickborne bunyavirus. Of 25 patients hospitalized with this disease in China, 100% produced and maintained neutralizing antibodies to severe fever with thrombocytopenia syndrome virus for the study period of 4 years.

Severe fever with thrombocytopenia syndrome (SFTS) is an emerging hemorrhagic fever disease in eastern Asia (*1**–**3*). SFTS is caused by SFTS virus (SFTSV), a tickborne bunyavirus that is transmitted through tick bites (*1**,**3**,**4*) and person to person by contact with patient blood (*5**,**6*). Clinically, SFTS is characterized by a sudden onset of fever, thrombocytopenia, hemorrhagic tendency, gastrointestinal symptoms, and multiple organ dysfunction and a high case-fatality rate of 12%–30% (*1*). SFTSV is a relatively new bunyavirus, and information regarding its neutralizing antibodies is scarce. In this study, we determined the prevalence and duration of neutralizing antibodies in serum samples of SFTS patients in China.

## The Study

For this study, patients with a clinical diagnosis of SFTS were those who had fever, thrombocytopenia, or leukopenia without another known acute infectious disease; patients with laboratory-confirmed SFTS had SFTSV antibodies or RNA detected by ELISA or reverse transcription PCR (RT-PCR). Acute-phase (within 2 weeks after onset of illness) and convalescent-phase serum samples obtained during hospitalization of the patients were tested for total antibodies against SFTSV by using a double-antigen sandwich ELISA kit (Xinlianxin Biomedical Technology Limited, Wuxi, China). The study was approved by the ethics committee of Shandong University. Informed consent was obtained from all participants.

The ELISA plates were coated with recombinant SFTSV nucleoprotein (*7*). Undiluted patient serum samples were used for ELISA; SFTSV antibodies were detected with horseradish peroxidase–labeled recombinant SFTSV protein. Serum samples were considered positive for SFTSV when absorbance of the sample was >2.1 times that of a negative control at 450 nm (*8*). Nested RT-PCR amplification of the SFTSV RNA large segment (900 bp) and small segment (600 bp) have been described previously (*8*). PCR products were confirmed to be SFTSV RNA by DNA sequencing.

During June 26, 2011–August 26, 2012, a total of 46 patients were hospitalized and given a clinical diagnosis of SFTS in a local hospital in Yiyuan County, Shandong Province, China. We confirmed by ELISA or RT-PCR that 33 (71.7%) of these 46 patients were infected with SFTSV. Of the confirmed cases of SFTS, 22 occurred in 2011 and were reported previously (*8*). Two (6.1%) patients with confirmed SFTS died.

Among the 31 laboratory-confirmed living patients with SFTS, 25 agreed and 6 refused to donate blood samples for neutralization assay after discharge. Thirteen (52%) volunteers were male and 12 (48%) were female; their ages ranged from 42 to 75 years (median age 62 years).

Blood samples were obtained from the 25 SFTS volunteers 2 or 3 times during a 4-year period. Serum samples were heat inactivated at 56°C for 30 min and diluted in 2-fold increments from 1:5 to 1:1,280. Each dilution of serum was mixed with an equal volume of solution containing SFTSV (1,000 pfu/mL) at 37°C for 1 hour. Culture medium was used as a control for serum. Samples were tested by using the 50% plaque reduction neutralization test (PRNT_50_). The titer obtained is the reciprocal of the highest serum dilution that reduces the number of plaques by 50% relative to the average number of plaques in viral control wells.

At first, SFTSV does not form clear plaques on Vero cells. SFTSV is passaged on Vero cells until plaques are clearly visible. Initially, 10^6^ SFTSV is inoculated into cells in 1 well of a 6-well plate. When a cytopathic effect is visible, cells with the cytopathic effect are aspirated with a pipette tip and transferred to a new well. A single plaque is picked and used for viral stock when the plaques are clearly visible on the fifth passage.

To determine the viral titer with a plaque assay, the viral stock is diluted from 10^−2^ to 10^−6^ in 10-fold increments. Each dilution of viral stock is used to infect 2 wells of cells. (The negative control contains maintenance medium without virus.) Infected cells are incubated at 37°C in 5% CO_2_ for 1 h; then, viral inoculum is replaced with Dulbecco’s modified Eagle medium containing 1.5% methylcellulose, 1% fetal bovine serum, 10 mmol/L HEPES, penicillin (100 units/mL), and streptomycin (100 µg/mL). Plates are incubated at 37°C in 5% CO_2_ for 10 d. The monolayer is fixed with 4% paraformaldehyde and stained with crystal violet. Plaques in each well are counted to determine the plaque-forming unit.

PRNT_50_ results showed that all 25 patients developed neutralizing antibodies against SFTSV at titers from 20 to 640; the neutralizing antibodies lasted for the entire study period of 4 years (Table). We also performed PRNT_90_ tests for all 25 patients; these showed similar results to PRNT_50_, but the titers were less in extent than those of PRNT_50_ (data not shown). 

**Table:** Neutralizing antibody titers for convalescent-phase serum samples from 25 patients with SFTS, Yiyuan County, Shandong, China, 2011–2014*

Patient no.	Titer by PRNT_50_			
First year	Second year	Third year	Fourth year
1	640	NT	320	80
2	80	NT	40	80
3	NT	NT	40	40
4	20	NT	40	40
5	20	NT	40	40
6	80	NT	80	20
7	40	NT	80	40
8	5	NT	20	40
9	40	NT	40	20
10	160	NT	80	80
11	160	NT	20	80
12	160	NT	40	40
13	40	NT	20	NT
14	NT	NT	320	NT
15	160	NT	160	80
16	320	NT	40	40
17	160	NT	40	40
18	NT	NT	80	40
19	640	NT	160	160
20	320	80	80	NT
21	640	320	80	NT
22	20	20	20	NT
23	320	80	NT	NT
24	NT	80	40	NT
25	640	80	160	NT

*Convalescent-phase serum samples were obtained during the first, second, third, and fourth years after discharge of the patients from the hospital. NT, no test performed because serum was not available; PRNT_50_, plaque reduction neutralization test indicating the serum titer that reduced 50% of plaque-forming units of SFTS virus; SFTS, severe fever with thrombocytopenia syndrome.

In general, the titer of neutralizing antibodies decreased over time in all but 2 patients (nos. 5 and 11), who had a higher PRNT_50_ titer in the last year than in the first year; this increase may have been caused by reinfection with SFTSV. However, our previous studies indicate that, in the local area, the incidence of SFTSV infection is <5 cases/100,000 population and the seroprevalence rate of SFTSV in the healthy population is <1% (*8**–**10*), suggesting that the chance of reinfection of a patient with SFTSV is low. We cannot exclude, however, that these 2 patients could have been infected with other phleboviruses that have not yet been isolated in China. Serum samples from 2 healthy persons were also tested for neutralizing antibodies as controls; neither of them had any neutralization activity against SFTSV.

## Conclusions

We found that hospitalized patients with SFTS produced long-lasting neutralizing antibodies to SFTSV. We do not know the characteristics of the neutralizing antibodies against SFTSV, which need to be further investigated. In general, neutralizing antigens of bunyavirus are located on the viral glycoproteins (*11**–**13*). A neutralizing monoclonal antibody to SFTSV is found to bind a linear epitope in the ectodomain of glycoprotein Gn of SFTSV. Its neutralizing activity is attributed to blockage of the interactions between the Gn protein and the cellular receptor (*14*). The limitation of this study is that we obtained SFTS patients’ serum samples for only up to 4 years after diagnosis; we do not know how long the neutralizing antibodies in patients will last.
